# Improved methods for mass production of magnetosomes and applications: a review

**DOI:** 10.1186/s12934-020-01455-5

**Published:** 2020-10-20

**Authors:** Abdul Basit, Jiaojiao Wang, Fangfang Guo, Wei Niu, Wei Jiang

**Affiliations:** 1 State Key Laboratory of Agro-Biotechnology, College of Biological Sciences, China Agricultural University, Beijing, 100193 China; 2 Department of Microbiology, Faculty of Life Sciences, University of Okara, Okara, 56130 Pakistan; 3 Beijing Key Laboratory for Prevention and Control of Infectious Diseases in Livestock and Poultry, Institute of Animal Husbandry and Veterinary Medicine, Beijing Academy of Agriculture and Forestry Sciences, Beijing, BJ People’s Republic of China

**Keywords:** Magnetosome biosynthesis, high-yield, cell growth, culture conditions, commercial applications

## Abstract

Magnetotactic bacteria have the unique ability to synthesize magnetosomes (nano-sized magnetite or greigite crystals arranged in chain-like structures) in a variety of shapes and sizes. The chain alignment of magnetosomes enables magnetotactic bacteria to sense and orient themselves along geomagnetic fields. There is steadily increasing demand for magnetosomes in the areas of biotechnology, biomedicine, and environmental protection. Practical difficulties in cultivating magnetotactic bacteria and achieving consistent, high-yield magnetosome production under artificial environmental conditions have presented an obstacle to successful development of magnetosome applications in commercial areas. Here, we review information on magnetosome biosynthesis and strategies for enhancement of bacterial cell growth and magnetosome formation, and implications for improvement of magnetosome yield on a laboratory scale and mass-production (commercial or industrial) scale.

## Introduction

The research interest of nanoparticles applications in the fields of biomedical, biotechnology and environmental protection has gained tremendous importance in recent years. The development of magnetic nanoparticles is the outcome of that investigative focus and significance [1]. The physico-chemical procedures for the preparation of magnetic nanoparticles involve high cost and chemicals with environmental implications and human health hazards. Thus, the need arises to ascertain and use environmental friendly, biocompatible, cheap, and low energy demanding methods for preparation of nano-particles [2]. In this situation, the nano-particles synthesized by magnetotactic bacteria with distinctive characteristics would be right choice and requisite for biomedical and biotechnology applications [3]. Magnetosomes, which consist of membrane-enveloped, nano-sized magnetite (Fe_3_O_4_) or greigite (Fe_3_S_4_) crystals arranged in chain-like structures, are synthesized by a variety of magnetotactic bacteria [4]. Many recent studies have involved cultured magnetotactic bacterial strains, particularly *Magnetospirillum gryphiswaldense* MSR-1, *Magnetospirillum magneticum* AMB-1, *Magnetospirillum magnetotacticum* MS-1 and *Magnetospirillum* sp. ME-1 [5, 6]. Investigations of these strains have greatly increased our understanding of molecular mechanisms of magnetotactic bacteria in general [5]. Chemical composition, morphology, and size of magnetite crystals are uniform within a given strain, but differ among strains of magnetotactic bacteria. Magnetite and greigite crystals of magnetosomes generally display constant shape, although slight variations of size and shape are sometimes observed for greigite [7].

Considerable research effort has therefore been focused on production of high-quantity magnetosomes [8, 9]. However, their cultivation under experimental conditions has been difficult because of their highly precise and restricted living conditions [10]. Isolation, identification, and characterization of magnetosomes are generally at an early research stage. The major obstacle to mass-production (commercial or industrial) scale cultivation/ growth of magnetotactic bacteria (MSR-1, AMB-1, MS-1 and ME-1) is the need for high magnetosome yield at reasonable expense and energy cost [11, 12]. In theory, magnetosome yield can be improved through modification (optimization) of culture medium composition and growth conditions [12, 13]. However, few studies have focused on such optimization. Effects on growth of changes in factors such as temperature, pH, dissolved oxygen concentration, and concentrations of various salts and acids have repeatedly been investigated, but greater emphasis is needed on maximization of magnetosome yield [12]. Here, we review information on magnetosome biosynthesis and strategies used for enhancement of bacterial cell growth and magnetosome formation, and implications for improvement of magnetosome yield on a laboratory scale and mass-production (commercial or industrial) scale.

## Mass production of magnetosomes

**Fig. 1:**
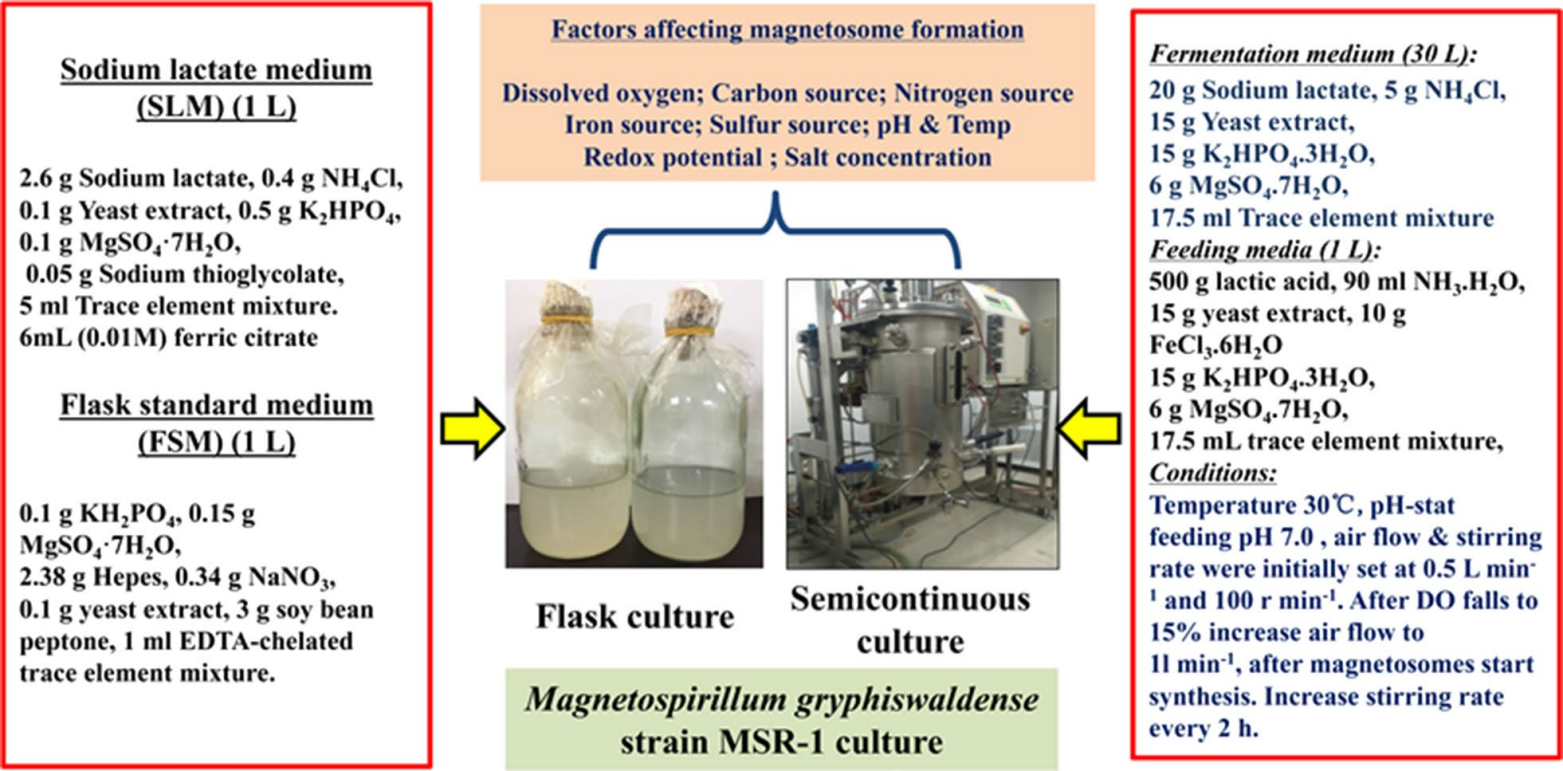
Culture media used for cultivation of *M. gryphiswaldense* strain MSR-1, and factors that affect MSR-1 growth [15]

Mass production of magnetosomes for commercial applications remains a challenging task. Cultivation of magnetotactic bacteria (MSR-1, AMB-1, MS-1) is difficult because of their diverse metabolisms, although several types of culture media have been developed for high magnetosome yield [14]. MSR-1 has been utilized extensively as a model microorganism for studies of magnetosome formation. Growth conditions affect the physical properties of magnetosomes synthesized by MSR-1. In particular, depending on the bacterial growth phase (either at the logarithmic phase or at the stationary phase), the magnetosomes present differences in size and stoichiometry [8]. Optimal culture conditions (in regard to dissolved oxygen concentration, pH, nutrient composition, and salt concentrations) are highly restrictive. A variety of control strategies have been evaluated for achieving more efficient magnetosome yield. Optimized culture conditions for growth of MSR-1, based on studies to date, are summarized in Fig. 1.

## Factors affecting the mass production of magnetosomes

### Nutrient-balanced feeding

The major factor affecting growth of magnetotactic bacteria, and consequently magnetosome formation, is concentration of nutrients, particularly carbon source. It was not possible to extend optimized medium conditions determined for MSR-1 growth in shake-flask culture directly to mass-production scale fermentor culture [15]. During MSR-1 culture, accumulation of excessive nutrients and inhibitory components in medium exerts rate-limiting effects on cell growth. According to Liebig’s Law of the Minimum, biomass in a given system is typically restricted by the amount of one particular nutrient, even when other nutrients are present in excess [16].

A nutrient-balanced feeding strategy can reduce the inhibitory effect of excessive amount of nutrients in medium. In this strategy, accumulation of Na^+^ and Cl^−^ ions is reduced by replacement of carbon and nitrogen sources. In fed-batch culture, accumulation of Na^+^ and Cl^−^ ions decreases osmotic potential and consequently inhibits cell growth. Even a low NaCl concentration (40 mM [2.34 g L^− 1^]) inhibited cell growth [17]. Thus, a nutrient-balanced feeding strategy can significantly enhance growth rate.

Liu et al. [15], established a “chemostat culture” technique for MSR-1 cultivation based on pH-stat feeding to maintain consistency of nitrogen, carbon, and iron concentrations using several organic acids. Microaerobic conditions were applied for MSR-1 cultivation in a fed-batch autofermentor system. A nutrient solution containing (per liter) ferric citrate (4.2 g), lactic acid (52.6 g), sodium lactate (129 g), and NH_4_Cl (54.9 g) was used for pH-stat feeding. High values of magnetosome yield (83.23 ± 5.36 mg L^− 1^) and cell growth (55.49 mg L^− 1^ day^− 1^) were achieved at low sodium lactate level (Table 1). Chemostat culture technique efficiently promotes magnetosome yield and cell growth with low time and energy cost. Cytotoxic effects were observed for excessive dissolved oxygen concentration (≥ 20 ppb) and presence of lactic acid in medium. Artificial control strategies for autofermentor systems must be adjusted in regard to physiological condition of cells. Similarly, Fernández-Castané et al. [18], demonstrated the pH-stat fed-batch growth strategy. In this strategy various concentrations of the lactic acid (carbon source) and sodium nitrate (electron acceptor) were applied in the feed. Growth conditions and intracellular iron concentration were optimized according to the biomass concentration. The highest biomass concentration reached to OD_565__nm_ = 15.50 [18].

**Table 1:** Yield production of magnetosomes by magnetotaic bacteria and their conditions

Strains	Growth Media	pH and Temperature	Dissolved oxygen	Yield production	References
ME-1	OFM	7.0 and 30 °C	0.5 ppm	120 mg L^− 1^	[6]
MS-1	MSGM + PEG	7.0 and 28 °C	1%	21.8 mg L^− 1^	[11]
MSR-1	OFM	6.8 and 30 °C	0 ppm	83.23 ± 5.36 mg L^− 1^	[15]
MSR-1	OFM (feed batch)	6.9 and 30 °C	0–1 ppm	225.53 mg L^− 1^	[17]
MSR-1	OFM (semi-continuous)	6.9 and 30 °C	0–1 ppm	168.3 mg L^− 1^ (dry weight basis)	[17]
AMB-1	MSGM	6.7 and 25 °C	–	7.5 mg L^− 1^	[19]
MSR-1	OCM	6.8 and 30 °C	5–10 ppm	186.67 mg L^− 1^	[23]
AMB-1	LSM	7.0 and 28 °C	0.25 mbar	3.3 mg L^− 1^	[25]
MSR-1	LSM	7.0 and 28 °C	0.25 mbar	6.3 mg L^− 1^	[25]
MSR-1	OFM	7.0 and 30 °C	0.5%	58.4 ± 6.4 mg L^− 1^	[31]
AMB-1	MSGM	6.7 and 26 °C	–	4.5 mg L^− 1^	[90]
MSR-1	OGM	6.8 and 30 °C	> 1 ppm	26 ± 3 Magnetosomes no.	[91]

*OFM* optimized flask medium, *OCM* optimized culture medium, *MSGM* *Magnetic Spirillum* growth medium, *LSM* large scale medium, *OGM* optimized growth medium, *PEG* polyethylene glycol

MSR-1 cell growth and magnetosome formation are high when sodium lactate is used as carbon source. On the other hand, low sodium lactate concentration is needed to maintain low dissolved oxygen concentration for rapid cell growth and magnetosome formation [15]. Maintaining sodium lactate concentration in mass production scale-up is difficult, and specific feeding strategies are therefore required in the laboratory. NH_4_Cl has been shown to be a better nitrogen source than NaNO_3_ [15, 17].

Zhang et al. [17], achieved maximal magnetosome yield in MSR-1 using a semi-continuous culture strategy. Optimized flask medium was used in 7.5- and 42-L autofermentors, nutrient-balanced feeding strategy was applied, and carbon and nitrogen sources were replaced to reduce accumulation of Na^+^ and Cl^−^ ions. Osmotic potential was decreased by Na^+^ and Cl^−^ ion accumulation, thereby inhibiting magnetosome yield and cell growth. We achieved maximal values in fed-batch culture of magnetosome yield 356.52 mg L^− 1^ and cell growth 9.16 g L^− 1^ [17] (Table 1).

Yang et al. [19], cultured AMB-1 cells in magnetic spirillum growth medium (MSGM) enriched with L-cysteine, yeast extract, and polypeptone. In this system, L-cysteine enhanced cell growth and reduced lag phase, resulting in high magnetosome production. The addition of only yeast extract and polypeptone results in slightly production of magnetosomes. Yeast extract displays no significant effect in magnetosome production, whereas polypeptone only increases the final cell density [20]. The reason for improved production of magnetosomes by L-cysteine is unknown, however, it is assumed that magnetosome production is not associated with lower redox potentials in the presence of L-cysteine [20]. Moreover, AMB-1 can grow without available amino acids and L-cysteine synthesis pathways in AMB-1 may be not efficient or related to cell growth. Therefore, AMB-1 may directly use L-cysteine instead of having to synthesize it for facilitating cell growth [20].

Ke et al. [6], cultured *Magnetospirillum* sp. ME-1 in growth medium enriched with sodium acetate, sodium succinate, yeast extract, MgSO_4_, NH_4_Cl and ferric citrate. ME-1 utilizes carbon source for growth such as succinate, fumarate, oxaloacetate, pyruvate, acetate, lactate, malate and peptone. In addition, ME-1 can grow in the absence of nitrogen source, however, NH_4_Cl or NaNO_3_ supplementation enhance the ME-1 growth. ME-1 exhibit urease and oxidase activity, suggesting the capability of aerobic growth, however, aerobic condition inhibits the magnetosome formation in ME-1 [6]. Fed-batch fermentation of ME-1 was optimized at a constant level of pH 6.8 in a 10-L fermenter based on pH-static feeding, while supplying the carbon, nitrogen and iron sources for large-scale production [6] (Table 1).

Despite the high yield of magnetosomes, such developed methods for the growth of magnetotactic bacteria contains toxic components in growth medium. These components include carcinogenic, mutagenic and reprotoxic chemicals, heavy metals, chelating agents and un characterized animal derived ingredients such as yeast extract [21]. There is a great need to obtain large scale production of pure magnetosomes with lowest possible amount of such impurities or toxic components (other metals than iron). Therefore, Berny et al. [22], developed a minimal growth medium for magnetosomes production with less or devoid of toxic components, and having similar magnetosome properties as those obtained in the best reported growth conditions by Zhang et al. [17]. Firstly, magnetotactic bacteria were amplified in pre-growth medium [containing sodium lactate (2.6 g), NH_4_Cl (0.4 g), MgSO_4_·7H_2_O (0.1 g), K_2_HPO_4_ (0.1 g), thiamin HCl (40 µg), ME6 (0.5 mL)] without producing magnetosomes [22]. In second step, magnetotactic bacteria were then fed with an iron rich fed-batch medium containing [sodium lactate (1.3 g), NH_4_Cl (0.2 g), MgSO_4_·7H_2_O (0.03 g), K_2_HPO_4_ (0.03 g), thiamin HCl (27 µg), of ME6 (0.08 mL)] to allow magnetosome synthesis [22]. After 50 h of growth, biomass concentration reached to OD_565__nm_ = 8 and yield magnetosomes production of about 10 mg/L of growth medium. A significant reduction/disappearance in magnetosome composition of Zn, Mn, Ba, and Al were observed [22]. This new strategy for the magnetosomes production without or lowest concentration of impurities other than iron, paves the way towards medical applications.

### Dissolved oxygen concentration

Magnetosome biosynthesis requires microaerobic or anoxic conditions. Low dissolved oxygen level significantly affects cell growth because high-density culture requires high dissolved oxygen to obtain desired magnetosome yield. On the other hand, increased dissolved oxygen may increase MSR-1 density in culture medium but inhibit magnetosome formation [15]. A conflict thus exists between magnetosome formation and cell growth, making it difficult to simultaneously achieve high MSR-1 cell density and high magnetosome yield. This conflict may be resolved somewhat by controlling dissolved oxygen to an optimal level through adjustment of cell growth rate. Jajan et al. [23], reported the reduced iron uptake and magnetosomes production at the dissolved oxygen level of above 5–10 ppm. However, when dissolved oxygen was lower than 5–10 ppm, iron uptake rate and production of magnetosomes was increased, which probably due to the slow growth of bacteria [23]. Sun et al. [24], established mass culture of MSR-1 for enhanced magnetosome production in a 42-L fermentor, with optimized flask medium, by applying strict microaerobic conditions (near-zero dissolved oxygen concentration) and using ferric citrate and sodium lactate as iron and carbon sources in medium. This strategy was effective for yield cultivation of magnetosomes because cell growth was regulated at low dissolved oxygen concentration, resulting in high magnetosome yield.

AMB-1 is a facultative anaerobic magnetotactic bacterium that transfers electrons via two respiratory pathways. Under aerobic growth conditions, AMB-1 utilizes oxygen as electron acceptor and neither promotes nor inhibits formation of magnetic particles. In an alternative pathway, AMB-1 uses nitrate as electron acceptor and therefore requires low media redox potentials, which are conducive to magnetosome formation. In a study by Yang et al. [20], magnetosome production rate was high under low dissolved oxygen concentration in liquid phase. When dissolved oxygen concentration in liquid phase exceeded a certain level (0.20 ppm), the respiratory pathway shifted to aerobic growth, leading to reduced magnetosome production.

Dissolved oxygen concentration is strongly affected by air flow rate and stirring rate. When dissolved oxygen during initial growth phase is raised by increasing air flow and stirring rates, magnetosome yield remains low until dissolved oxygen declines to an undetectable level. To overcome this phenomenon in MSR-1 cultivation, dissolved oxygen must be enhanced to an optimal level by stirring, and cells then allowed to reduce dissolved oxygen through respiration, until reaching the level optimal for magnetosome formation. High magnetosome production was achieved by optimizing/ adjusting air flow and stirring rates [15]. During initial culture phase, dissolved oxygen was reduced by maintaining these rates respectively at 1 L min^− 1^ and 200 rpm min^− 1^. During later culture phase, dissolved oxygen was increased by adjusting air flow rate to 2 L min^− 1^ at 20 h and stirring rate to 300 rpm min^− 1^ at 28 h. Under these conditions, cells grew rapidly, dissolved oxygen become undetectable at 12 h, and cell density reached OD_565__nm_ = 12.3 at 36 h. Sodium lactate and ferric citrate concentrations were controlled respectively in the ranges 3–6 mmol L^− 1^ and 70–110 µmol L^− 1^ during the process. High values of magnetosome yield (83.23 ± 5.36 mg L^− 1^) and productivity (55.49 mg L^− 1^ day^− 1^) were thus attained [15] (Table 1).

In ME-1 cultivation, dissolved oxygen was controlled to enhance the magnetosome production at a constant level of 0.5% by coupling to the air-flow rate and stirring rate. During fed-batch fermentation, a stirring rate (in the range of 50–300 rpm) produced a large amount of magnetosomes at constant level of dissolved oxygen (0.5%). The resulting cell density and magnetosome yield at 49 h were 6.5 (OD_565_) and 120 mg L^− 1^ (wet weight). This strategy attained high magnetosome yield and productivity, thus indicate that ME-1 has great potential for the large-scale production of magnetosomes [6].

Low dissolved oxygen levels have been established empirically in many studies, but without continuous measurement of dissolved oxygen concentration or definition of its control in the medium. Heyen and Schüler [25], established a method for automatic control of low oxygen tension (pO_2_) in MSR-1 culture medium using a fermentor system for oxystat operation. pO_2_ tension was correlated with magnetite formation. The lowest recorded pO_2_ value (0.25 mbar; 1 bar = 10^5^ Pa) was the most favorable for magnetosome formation. Cells grown under oxystat conditions showed significantly higher magnetite yield (6.3 mg L^− 1^ day^− 1^) (Table 1).

### Ferric ion uptake

Iron is required as a cofactor for many enzymes, particularly those involved in major biological pathways. Specific iron transport mechanisms in cells provide iron levels sufficient for optimal growth. Some bacteria produce ferric chelators (termed siderophores) to take up ferric iron (Fe^3+^). Magnetotactic bacteria synthesize magnetosomes composed of magnetite or greigite after finding microaerophilic conditions suitable for their growth [26]. In MSR-1, magnetite is the major component of magnetosomes, and magnetosome production is therefore not significantly affected by ferric citrate concentration in culture medium. Jajan et al. [23], showed that ferrous sulfate was a better source of iron than ferric quinate and ferric citrate for *M. gryphiswaldense* [23]. In a study of AMB-1, Yang et al. [19], used various ferrous sulfate and ferric chelates as iron sources, and compared their effects. Magnetosome production was significantly enhanced by ferric gallate and sulfate, and was also affected by other iron source (ferric quinate, ferric malate), and by iron uptake rate.

Ferric ion (Fe^3+^) is taken up during dynamic cell growth, and the amount taken up is correlated with magnetosome formation when dissolved oxygen level in medium is undetectable. Magnetosome formation requires micromolar iron concentration and microoxic conditions [27]. MSR-1 cells are nonmagnetic under oxic conditions, but begin to produce magnetite when dissolved oxygen concentration falls below a threshold value (20 mbar or undetectable). In log phase of cell growth, ferric ion is taken up rapidly, and its absorption rate is > 80% and correlated with magnetosome formation [15].

### Magnetosome synthesis consumes ATP

ATP is the universal energy source required for metabolism, molecular transportation, signal transduction, and other crucial cell physiological processes. Magnetosome synthesis requires a large amount of energy, and iron uptake depends on ATP availability. NADH provides a proton gradient across the inner mitochondrial membrane for ATP production catalyzed by ATP synthase [28]. NADH/NAD^+^ ratio increases rapidly following magnetosome maturation during log phase.

Reducing power increases significantly during magnetosome synthesis; however, excessive reducing power may inhibit magnetosome synthesis and cell growth [17]. MSR-1 can consume excessive reducing power through polyhydroxybutyrate (PHB) synthesis and hydrogen release [29]. MSR-1 cells contain PHB granules [30]. Knockout of PHB synthase gene in MSR-1 resulted in ~ 30% increase of magnetosome number [31]. Energy competition thus occurs between PHB and the magnetosome synthesis process. A mutant of ATPase gene of MSR and MSR-NPHB created by conjugation was used as a genetic engineering tool to demonstrate that chloramphenicol acetyltransferase (CAT) promoter increases downstream expression of ATPase gene. In comparison with MSR-1, MSR-NPHB displayed 35% greater hydrolysis activity, 71% lower PHB accumulation, 56% greater oxygen consumption, and 40% greater lactate consumption. Maximal yield of MSR-NPHB in a 7.5-L bioreactor was 58.4 ± 6.4 mg L^− 1^ [31]. These findings demonstrate that magnetosome yield can be enhanced, and production cost and time reduced, through genetic manipulation of MSR-1 in combination with optimization/ modification of culture and growth media.

### Superoxide dismutase activity

Magnetosome synthesis is associated with *in vitro* breakdown of H_2_O_2_, and with protective effects against H_2_O_2_ toxicity in cells. In microorganisms, the enzyme superoxide dismutase breaks down H_2_O_2_ and superoxide anion radical (O_2_^−^), both of which have destructive effects on cell macromolecules [32]. In magnetotactic bacteria, superoxide dismutase also reduces oxidative stress during magnetosome formation. H_2_O_2_ may form a hydroxyl radical after receiving an electron from ferrous iron (Fe^2+^). Hydroxyl radical is the reactive oxygen species (ROS) that can damage biomolecules [33]. Yang et al. [30], demonstrated that when sufficient dissolved oxygen and nutrients are available in late log phase, magnetosome formation and maturation are unable to catch up to cell division rate, leading to dilution of magnetosomes. Reduction of superoxide dismutase activity may thus result from similarity of diluted ROS. Magnetosomes, as well as artificial magnetic nanoparticles, participate in scavenging of ROS [27, 34], and this activity may also lead to reduced superoxide dismutase activity.

## Rapid large-scale purification of magnetosomes

**Fig. 2:**
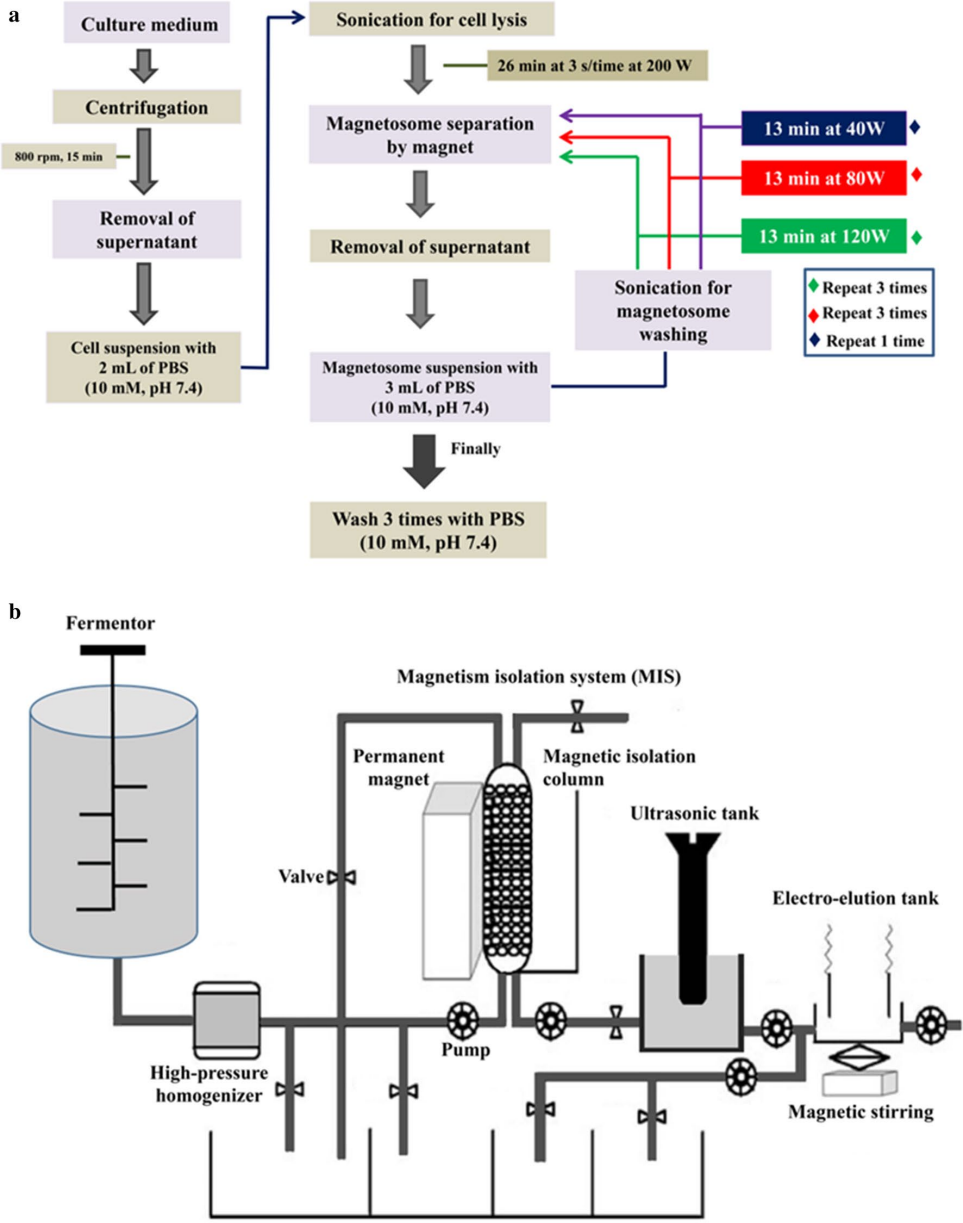
Two systems for extraction and purification of magnetosomes (see text)

Improved and optimized methods for extraction and purification of magnetosomes are needed, for reasons mentioned in Introduction. Magnetosome extraction methods have been described in numerous publications [35–37]. However, methods described to date for magnetosome purification, which involve disruption of cells by sonication, washing with PBS buffers, ultrasonic bathing, and collection of magnetosomes using a magnet (Fig. 2A), are not suitable for large-scale systems. Guo et al. [36], developed a rapid, continuous system for large-scale magnetosome purification. The system involved a high-pressure homogenizer, a magnetic isolation system (MIS), ultrasonic and electro-elution equipment, and magnetic stirring (Fig. 2B). The MIS consisted of an ultrasonic tank, a permanent magnet, and a magnetic isolation column. The magnetic isolation column was a plastic cylinder filled with iron beads (diameter 4–6 mm), with four interfaces (inlet or outlet) at the two ends. The two interfaces at the bottom end carried buffer and solution containing cells/ magnetosomes. The two interfaces at the top end carried buffer and discharged wastewater. The buffers used in the MIS were sterilized by heating at 121°C for 20 min, and the magnetic isolation column was cleaned before use by pumping through a 75% ethanol solution (v/v). The high-pressure homogenizer and MIS were prewetted with one bed-volume of PBS buffer. Culture solution mixed with buffer was used to lyse cells in the homogenizer, and cell extract was pumped into the MIS. Solution at controlled flow rate was passed through the homogenizer, lysed solution was pumped into the MIS, and waste residue was discharged through the outlet. Magnetosomes in the MIS were washed with six bed-volumes of buffer containing various concentrations of urea, and then washed into the ultrasonic tank by ultrasonication process. Urea solution in the ultrasonic tank was replaced by the buffer, magnetosomes were dispersed by ultrasonication, and buffer and magnetosome solution were simultaneously pumped back into the MIS by operating pumps. After binding of magnetosomes by magnetic force, six bed-volumes of buffer were pumped into the MIS. Ultrasonication was activated and magnet removed in the MIS such that magnetosomes were released and washed back into the ultrasonic tank. The above procedure was repeated three or four times until proteins in waste solution were undetectable at OD_280_.

The industrial development of magnetosomes has been raised by high yield magnetosome production methods. However, magnetotactic bacterium are Gram-negative bacteria, which contains bacterial endotoxin such lipo-polysaccharides [38], that probably effect magnetosomes applications. Therefore, several studies revealed the production of sterile or endotoxin free magnetosomes by removing of surrounding biological materials from magnetosomes mineral core [38, 39]. Since magnetosome formation results from the invagination of the bacterial membrane, the endotoxin concentration at the magnetosome surface might be higher than the tolerated concentration. Therefore, followed by the extraction and purification of magnetosomes from magnetotactic bacteria, organic materials such as endotoxins can be removed with the treatment of different detergents (SDS, Triton X100, phenol and chloroform) at 60°C in the presence of sonication [39]. The obtained uncoated magnetosome minerals can be characterized by transmission electron microscope (TEM). The detection and quantification of endotoxin concentration of uncoated magnetosome minerals can further be estimated by limulus amebocyte lysate (LAL) assay. Deduced endotoxin concentration can be further verified by *in vivo* experiments. Hamdous et al. [38], in their study, examined the endotoxin concentrations by administering the purified magnetosome suspension in the rabbits’ ear. The induction of any fever among rabbits was indicated the pyrogenic or non-pyrogenic suspensions.

### Applications

**Fig. 3:**
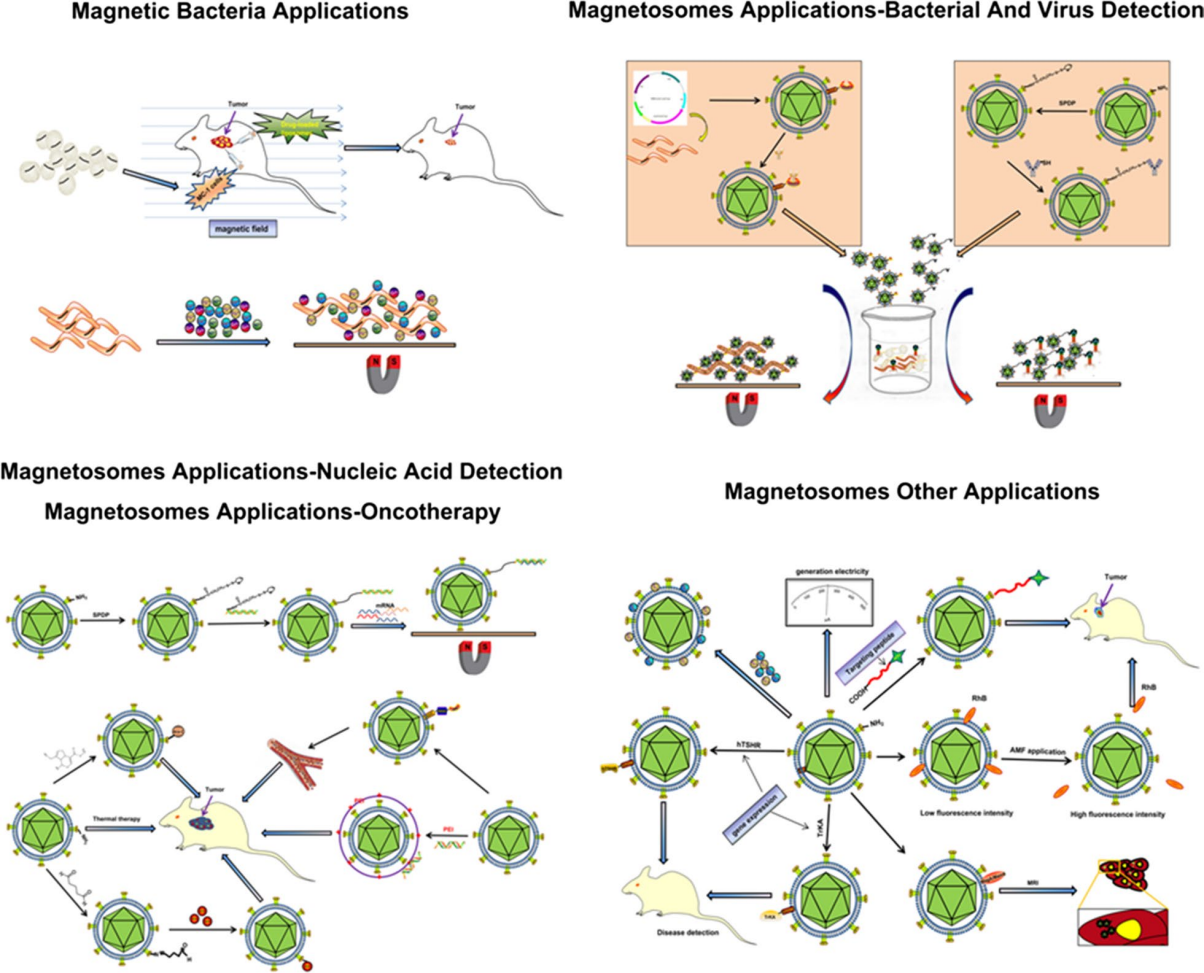
Schematic representation of magnetosomes applications

Magnetosomes are of considerable research and practical interest because of unique features such as narrow size distribution, uniform morphology, high chemical purity, low toxicity, and membrane envelope [37]. Applications of magnetosomes reflect their distribution, ferrimagnetic properties, nanoscale size, membrane-bound structure, and dispersal ability. Applications of magnetosomes are summarized in Fig. 3. Recent studies demonstrated that purified, sterilized MSR-1 magnetosomes are nontoxic to cancer cells *in vitro*, suggesting their potential application as drug carriers for clinical treatment of cancer and other diseases [35, 40–42]. Magnetosome membranes contain proteins and active groups that can function as binding sites. Synthetic magnetic nanoparticles utilize specific active surface groups, through either chemical coupling or physical adsorption. Because of the presence of a natural phospholipid membrane, coupling of magnetosomes with active substances such as chemotherapeutic drugs [37, 43] and peptides [40] is of great interest in biomedicine and drug delivery [44–50]. Magnetosomes wrapped with polyethyleneimine (PEI) can be used in gene delivery and cancer treatment [51–53]. Magnetosomes extracted from magnetotactic bacteria can be used as a magneto-thermal therapy for cancer treatment [54–56]. Other practical applications of magnetosomes includes as magnetic resonance imaging (MRI) probe or modified with protein for tumor detection [57, 58], the tracking of stem cells, dendritic cells and single nucleotide polymorphism (SNP) [59, 60], detection of pathogenic bacteria, viruses [61–63] and other immunodetection [64, 65], discrimination or recovery of DNA and mRNA [59, 66–68], adsorption and mineralization of metals [69–74] and known as the powerful tool for chip-based whole-cell biosensors [75]. “Surface display system” of magnetosomes used in immunoassay, enzyme reaction, and cell separation [76–87]. Magnetosomes can also be used as an effective model system to study CDF related II type diabetes [88]. Magnetotactic bacteria used to produce electricity and magnetic field induced rotation of magnetosome chains in silicified MTB [78, 89]. Thus, availability of high-quality magnetosomes will significantly increase potential applications, and it is therefore highly desirable and important to establish and develop rapid, simple, relatively inexpensive processes for yield cultivation of magnetosomes.

## Conclusions

The magnetosome production ability is of great interest in the areas of biotechnology, biomedicine, and environmental protection. The magnetosome formation is the outcome of the controlled biomineralization mechanisms and can be produced in different shapes and size ranges by magnetotatic bacteria. However, the need for more efficient cultivation methods for mass production of magnetosome represents the major obstacle to potential applications. For this purpose, various approaches have been developed and tested for such high-yield cultivation, including optimization of dissolved oxygen concentration, nutrient-balanced feeding strategies, and genetic engineering. Still, there remains an urgent need for further improved systems and optimization of conditions for enhancement of magnetosome yield at laboratory scale and mass-production (commercial or industrial) scale.

## Data Availability

Not applicable.
